# Radiation- and Photo-Induced Oxidation Pathways of Methionine in Model Peptide Backbone under Anoxic Conditions

**DOI:** 10.3390/ijms22094773

**Published:** 2021-04-30

**Authors:** Tomasz Pędzinski, Katarzyna Grzyb, Konrad Skotnicki, Piotr Filipiak, Krzysztof Bobrowski, Chryssostomos Chatgilialoglu, Bronislaw Marciniak

**Affiliations:** 1 Center for Advanced Technology, Adam Mickiewicz University, Uniwersytetu Poznanskiego 10, 61-614 Poznan, Poland; tomekp@amu.edu.pl (T.P.); piotrf@amu.edu.pl (P.F.); 2 Faculty of Chemistry, Adam Mickiewicz University, Uniwersytetu Poznanskiego 8, 61-614 Poznan, Poland; katgrz3@amu.edu.pl; 3 Institute of Nuclear Chemistry and Technology, Dorodna 16, 03-195 Warsaw, Poland; k.skotnicki@ichtj.waw.pl; 4 ISOF, Consiglio Nazionale delle Ricerche, Via P. Gobetti 101, 40129 Bologna, Italy

**Keywords:** methionine, oxidation, pulse and γ-radiolysis, laser flash and steady-state photolysis, free radicals, high-resolution MS/MS

## Abstract

Within the reactive oxygen species (ROS) generated by cellular metabolisms, hydroxyl radicals (HO^•^) play an important role, being the most aggressive towards biomolecules. The reactions of HO^•^ with methionine residues (Met) in peptides and proteins have been intensively studied, but some fundamental aspects remain unsolved. In the present study we examined the biomimetic model made of Ac-Met-OMe, as the simplest model peptide backbone, and of HO^•^ generated by ionizing radiation in aqueous solutions under anoxic conditions. We performed the identification and quantification of transient species by pulse radiolysis and of final products by LC-MS and high-resolution MS/MS after γ-radiolysis. By parallel photochemical experiments, using 3-carboxybenzophenone (CB) triplet with the model peptide, we compared the outcomes in terms of short-lived intermediates and stable product identification. The result is a detailed mechanistic scheme of Met oxidation by HO^•^, and by CB triplets allowed for assigning transient species to the pathways of products formation.

## 1. Introduction

The oxidation of methionine residues (Met) in peptides and proteins is a crucial reaction in the biological environment [1]. Reactions of both one- and two-electron oxidants with Met have been studied in some details. The reactive oxygen species (ROS) network, initiated from superoxide radical anion (O_2_^•−^) and nitric oxide (NO^•^), regulates numerous metabolic processes. The production of two-electron oxidants like H_2_O_2_, ONOO^−^ or HOCl involves the reaction with Met residues in a site-specific manner with formation *S* and *R* epimers of methionine sulfoxide, Met(O) [1]. Interestingly, the two epimeric forms of sulfoxide are repaired enzymatically by methionine sulfoxide reductase Msr-A and Msr-B, respectively [2,3]. Met residues in proteins are not only preserved against oxidative stress, but these transformations play an important role in cellular signaling processes [3,4].

**Scheme 1:**
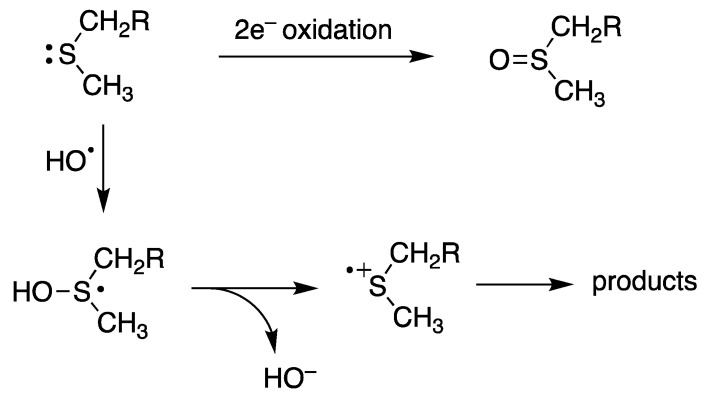
Methionine (R = CH_2_CH(NH_3_)^+^COO^−^) like thioethers reacts with two-electron oxidants to give the corresponding sulfoxide, while the reaction with HO^•^ affords other products.

Hydroxyl radicals (HO^•^) are the most reactive species within the ROS network and have long been regarded as a major source of cellular damage [5]. The main cellular processes that generate HO^•^ are the Fenton reaction of H_2_O_2_, the reduction of HOCl or H_2_O_2_ by O_2_^•−^ and the spontaneous decomposition of ONOOH [1]. In cells it is estimated that the diffusion distance of HO^•^ is very small due to its high reactivity with various types of biomolecules with rates close to diffusion-controlled [6,7]. Scheme 1 shows the two-step reaction of HO^•^ with sulfides like Met to give formally one-electron oxidation, i.e., the formation of sulfuranyl radical followed by heterolytic cleavage [8,9,10].

Radiolysis of water provides a very convenient source of hydroxyl radicals HO^•^. Time-resolved kinetic studies by pulse radiolysis have expanded our mechanistic understanding of radical reaction pathways of Met at various functionalized environment [11]. Indeed, neighboring group participation of one-electron oxidation of Met (MetS^•+^) reactivity within particular peptides and/or proteins is of great importance, due to the presence of a manifold of possible participating functionalities (carboxy, amine, hydroxy and amide groups) [12]. For a long time it was believed that the two-centered, three electron (2c-3e) bonds between the oxidized sulfur atom and the lone electron pairs, located on the nitrogen atom in the *N*-terminal amino group and the oxygen atoms in the *C*-terminal carboxyl group are responsible for the stabilization of MetS^•+^ through a five-membered or six-membered ring interaction [13,14,15,16]. Subsequent studies showed that heteroatoms present in the peptide bond can be also involved in the formation of similar transient species with 2c-3e bonds with the oxidized sulfur atom [14,15,16,17,18].

There are also a few radiation chemical studies of Met in aqueous solutions, followed by product characterization and quantification. In the reaction of HO^•^ with free Met in either the absence or the presence of oxygen, the attack at sulfur atom accounts of ~90% affording 3-methylthiopropionaldehyde; the formation of small amounts of the corresponding sulfoxide is due to in situ formation of H_2_O_2_ rather than to direct oxidation by HO^•^ [19,20]. Again, studying the reaction of HO^•^ with tripeptide Gly-Met-Gly provided strong evidence that the corresponding sulfoxide in the tripeptide derives from the in situ formed H_2_O_2_. It is relevant to say that the main product of the tripeptide is an unsymmetrical disulfide (RCH_2_SSCH_3_) assigned to the chemistry of MetS^•+^ with parent compound, while the use of aerobic conditions highlighted the formation of other products derived from peroxyl radicals of the tripeptide [17].

The present study focuses on the reaction of HO^•^ with Ac-Met-OMe (**1**), the simplest model peptide backbone. This reaction was previously studied by pulse radiolysis at the pH range 4–5.7 [14]. Herein we extend the identification and quantification of transient species by pulse radiolysis at pH 7 and of final products of γ-radiolysis by LC-MS and high-resolution MS/MS under anoxic conditions. The purpose of acetylation of the *N*-terminal amino group is to eliminate the fast intramolecular proton transfer from the amino group to the sulfuranyl moiety, which was suggested earlier as the main decay reaction pathway, while the esterification of the *C*-terminal carboxyl group eliminates its decarboxylation [8,9,17,21,22]. Therefore, the use of **1** allows to study the reaction of HO^•^ with Met residue with no contribution of *N*- and *C*-terminal functional groups.

**Scheme 2:**
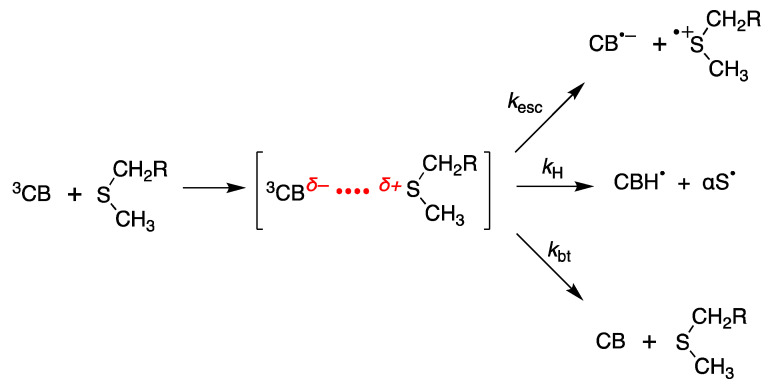
Known mechanism of primary photochemical reactions for sensitized photooxidation of methionine derivatives in aqueous solution.

Excited triplet states of benzophenones carboxyl derivatives (^3^CB) were also shown to be very useful for studying one-electron oxidation reactions of Met-containing molecules of biological significance [21,23,24]. The mechanism of primary photo-induced processes occurring during Met oxidation in aqueous solutions is summarized in Scheme 2.

The quenching of ^3^CB by Met derivatives leads to formation of a complex that can decay in three primary reactions: charge separation leading to CB^•−^ and MetS^•+^; H-atom transfer from alpha carbon atom next to sulfur to form CBH^•^ and αS^•^ radicals; and back electron transfer leading to formation of reactants in their ground states. Recently, the reaction of 3-carboxybenzophenone triplet (^3^CB) with Ac-Met-NHMe has been studied in some details by some of us [25]. The mechanism of photooxidation of Met moiety showed to involve mainly H-atom abstraction from the two α-positions next to sulfur (see Scheme 2). Herein we included results of CB sensitized photooxidation of Ac-Met-OMe (**1**) in aqueous solutions applying flash photolysis studies for transient detection and continuous photolysis for products identification.

Application at the same time of radiation and photochemical techniques allowed for the first time to have two complementary conditions in order to compare in detail the oxidation mechanisms of Met derivative **1** initiated either by HO^•^ or ^3^CB leading via short-lived intermediates to stable products.

## 2. Results and Discussion

### 2.1. Pulse Radiolysis Studies

Pulse irradiation of water leads to the primary reactive species e^−^_aq_, HO^•^, and H^•^, as shown in Reaction (1). The values in brackets represent the radiation chemical yield (*G*) in µmol J^−1^. In N_2_O-saturated solution (~0.02 M of N_2_O), e^−^_aq_ are efficiently transformed into HO^•^ radicals via Reaction (2) (*k* = 9.1 × 10^9^ M^−1^s^−1^), affording *G*(HO^•^) = 0.56 µmol J^−1^ [7].

(1) $$H_{2}O\;\to {\;e}_{\mathrm{aq}}^{-}\;\left(0.28\right),{\;\mathrm{HO}}^{•}\;\left(0.28\right),{\;H}^{•}\left(0.06\right)$$

(2) $$e_{\mathrm{aq}}^{-}+{\;N}_{2}O+{\;H}_{2}O\;\to {\;\mathrm{HO}}^{•}+{\;N}_{2}+{\;\mathrm{HO}}^{-}$$

**Figure 1:**
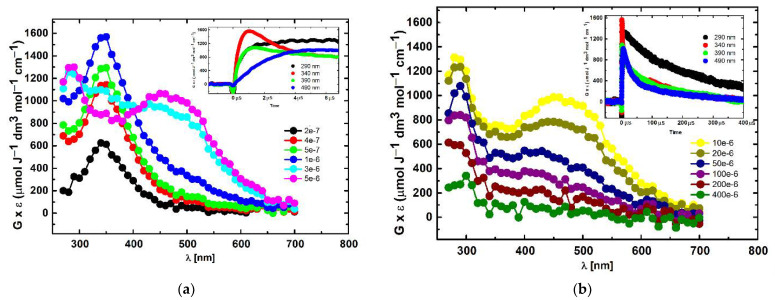
Absorption spectra following HO^•^ oxidation of compound **1** (0.2 mM) in N_2_O-saturated aqueous solutions at pH 7. Spectra were taken after the following time delays: (**a**) 200 ns (●), 400 ns (●), 500 ns (●), 1.1 μs (●), 3 μs (●), 5 μs (●) and inset: short-time profiles representing growths at *λ* = 290 nm (●), 340 nm (●), 390 nm (●) and 490 nm (●); (**b**) 10 μs (●), 20 μs (●), 50 μs (●), 100 μs (●), 200 μs (●), 400 μs (●) and inset: long-time profiles representing decays at *λ* = 290 nm (●), 340 nm (●), 390 nm (●) and 490 nm (●) (Dose per pulse = 11 Gy; optical path = 1 cm).

The reaction of HO^•^ with compound **1** was investigated in N_2_O-saturated solution of 0.2 mM **1** at natural pH (pH value of 7.0 was recorded). Transient absorption spectra in the range 270–700 nm recorded in the time range of 200 ns to 400 µs were collected in Figure 1.

**Scheme 3:**
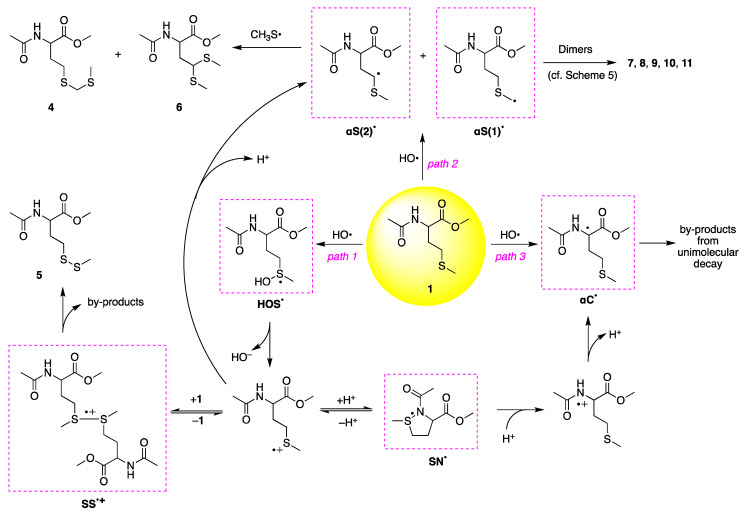
Proposed mechanism for the reaction of HO^•^ radicals generated by γ-irradiation of N_2_O-saturated aqueous solutions containing 1.0 mM *N*-acetyl methionine methyl ester (**1**) at natural pH.

The transient spectrum obtained 1.1 μs after the electron pulse showed a dominant sharp absorption band with *λ*_max_ = 340 nm. Based on previous studies on methionine derivatives, this band can be assigned to the HO^●^ adduct of the sulfur atom **HOS^●^** (cf. Scheme 3 for the structure) [13,14,17]. However, as shown in Figure 1, the transient spectrum profile changes with time, indicating the overlap of various transient species. The transient spectrum profile can be resolved into contributions from the following components: the sulfuranyl radicals (**HOS^●^**), the C_α_-centered radicals (**αC^●^**), the α-(alkylthio)alkyl radicals (**αS(1)^●^** and **αS(2)^●^**), the inter-molecular sulfur–sulfur radical cations (**SS^●+^**) and intramolecular sulfur-nitrogen three-electron-bonded radicals (**SN^●^**) (the structures of these intermediates are highlighted in colored boxes in Scheme 3; see also Figures S1 and S2 in Supplementary Materials for their individual spectra). These intermediates were previously identified during HO^●^-induced oxidation of **1**, but at the pH range 4–5.7 [14].

**Figure 2:**
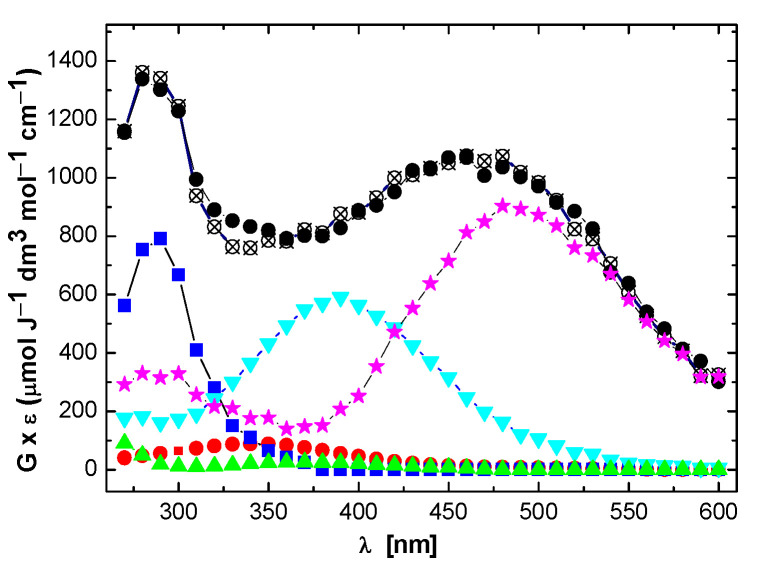
Resolution of the spectral components: HOS^●^ (●), αC^●^ (▲), αS(1)^●^ and αS(2)^●^ (■), SS^●+^ (★), SN^●^ (▼) in the transient absorption spectrum recorded 6 µs (●—experimental; ⊗—fit) after the electron pulse in N_2_O-saturated aqueous solution containing 0.2 mM **1** at pH 7.

The spectra recorded 1.1, 3 and 6 μs after the electron pulse were resolved into contributions from the same components (**HOS^●^**, **αC^●^**, **αS^●^**, **SS^●+^** and **SN^●^**). Figure 2 shows the spectrum at 6 μs, while Figures S3 and S4 report the spectra at 1.1 and 3 μs, respectively. The sum of all component spectra with their respective radiation chemical yields (*G*-values) resulted in a good fit (⊗ symbols in Figure 2, Figures S3 and S4) to the experimental spectra.

Table 1 reports the radiation chemical yields of each radical and their percentage contribution to the transient spectrum obtained.

The calculated total *G*-value of 0.53 μmol J^−1^ for the 1.1 μs spectrum is nearly in agreement with the expected *G*-value of HO^●^ (0.56 μmol J^−1^) available for the reaction of **1** at pH 7 and the concentration (0.2 mM) of **1**. This small difference in *G*-values could be understood, since at this time the reaction of HO^●^ radicals with **1** via pathways 2 and 3 (Scheme 3) is about to be completed. The spectrum showed a dominant sharp absorption band at *λ*_max_ = 340 nm. It is worthy to note that the most abundant radical present at this time is **HOS^●^**, which constitutes more than 70% of all radicals (Table 1). On the other hand, the total *G*-value of 0.56 µmol J^−1^ for 3 μs spectrum is in excellent agreement with the expected yield of HO^●^ radicals. The spectrum after 6 µs (Figure 2) was dominated by two distinct absorption bands with the pronounced maxima at *λ* ≈ 290 and 490 nm. These bands were assigned to **αS^●^** radicals and **SS^●+^** radical cations, respectively. The first one was obtained by hydrogen abstraction (path 2 in Scheme 3), and both of them by a sequence of reactions involving **HOS^●^** radicals and sulfur radical cations (S^●+^). The spectrum at 6 μs was resolved into contributions from the same components (Table 1). The total *G*-value of all radical present (0.57 µmol J^−1^) is again in excellent agreement with the expected yield of HO^●^ radicals. It is worthy to note that the most abundant radicals present at 6 μs are **αS^●^**, **SS^●+^** and **SN^●^** radicals which constitute more than 90% of all radicals. Interestingly, the comparison of the radiation chemical yields of **HOS^●^** radicals and the sum of radiation chemical yields of **αS^●^**, **SS^●+^** and **SN^●^** radicals at 3 µs and 6 µs after the pulse (Table 1) suggested that the increase of *G***(αS^●^** + **SS^●+^** + **SN^●^**) occurs at the expense of decrease of *G*(**HOS^●^**). This observation can be rationalized by the involvement of the S^●+^ on Met moiety in two equilibria (an acid-base and a concentration) and an irreversible deprotonation channel presented in Scheme 3.

Excellent material balance of all radicals, identified in the system equal to the *G*-value of HO^●^ radicals available for the reaction with **1**, proved the presence of all radical transients as the precursors of end products. At this point, it has to be stressed that after 6 µs the total *G*-value of the radicals began to decrease, reaching the value of 0.12 µmol J^−1^ at 400 µs which is only slightly higher than the G-value of **αS^●^** radicals (0.10 µmol J^−1^) (Figure S5). This suggests that on this time domain nearly 80% of all radicals formed in the system were consumed in radical-radical processes leading to the final products. Moreover, in this time domain the rates of these termination processes became dominant compared to the rates of transformation of **SS^●+^** and **SN^●^** radicals into **αS(1)^●^**, **αS(2)^●^** and **αC^●^** radicals, respectively.

Inset in Figure 1a shows kinetic traces recorded at four wavelengths (290, 340, 390 and 490 nm) that correspond to the maxima of absorption bands of the four most abundant radicals present in the irradiated system, i.e., **αS^●^**, **HOS^●^**, **SN^●^** and **SS^●+^**. These kinetic traces look different, and they reached their maximum signal at various times after the pulse. We assigned the observed buildup at *λ* = 340 nm to the formation of **HOS^●^** radical. Because **HOS^●^** is decaying in a significant way during its formation (see inset in Figure 1a), one has to account for this decay in order to get the proper value for the rate constant of the formation. Furthermore, based on the reference spectra applied in spectral resolutions (Figure S2), one can expect that the optical absorption bands of at least two radicals (**αC^●^** and **SN^●^**) overlap with the optical absorption band of **HOS^●^**, and thus may “contaminate” formation and decay traces observed at *λ* = 340 nm. In order to overcome this problem, we extracted the concentration profile of **HOS^●^** using spectral resolution of spectra at any desired time delay following the electron pulse ranged from 200 ns to 8 µs (cf. Figure S6A). Similarly, on the basis of the extracted concentration profiles of the other transients (**SS^●+^**, **αS^●^**, **αC^●^** and **SN^●^**), it was possible to evaluate their kinetic parameters (cf. Figure S6B–E). Otherwise, this would not be possible based just on “raw” time profiles recorded at the wavelengths of their absorption maxima (see insets in Figure 1). Kinetic parameters of all radical transients are collected in Table 2.

The first-order decay of **HOS^●^** (*k_d_* = 5.6 × 10^5^ s^−1^) results in radicals with a sulfur radical cationic site (S^●+^). The S^●+^ undergo typical reactions expected for such kind of radicals: deprotonation leading to the **αS^●^** radicals, the intermolecular formation of **SS^●+^** and intramolecular five-membered cyclic **SN^●^** (Scheme 3).

For the equilibrium S^●+^ + **1** ⇆ **SS^●+^**, the values of *k*_f_ = 2.2 × 10^9^ M^−1^s^−1^ and *k*_r_ = 3.6 × 10^4^ s^−1^ give the equilibrium constant *K* = *k*_f_/*k*_r_ = 6.1 × 10^4^ M^−1^, which is three-fold lower than the previously estimated *K* for analogous dimeric radical cations derived from (CH_3_)_2_S [26].

The formation of **αS^●^** radicals occur via two different mono-exponential processes with *k* = 4.1 × 10^5^ s^−1^ and *k* = 3.6 × 10^4^ s^−1^ (cf. Figure S6C). The first one is assigned to deprotonation of S^●+^ that is formed directly from **HOS^●^**, and the second to deprotonation of S^●+^ that is formed via the reverse reaction involving **SS^●+^**. These assignments are strongly supported by the fact that the rate constant of the slow formation of **αS^●^** radicals (cf. Figure S6C) is equal to the rate constant of the **SS^●+^** decay (cf. Figure S6B).

A second-order rate constant of 7.5 × 10^9^ M^−1^s^−1^ was obtained for the reaction of HO^●^ radical with **1**, via path 3 (Scheme 3), by measuring the rate constant of the pseudo-first-order growth *k* = 1.5 × 10^6^ s^−1^ of the **αC^●^** concentration at 0.2 mM of **1** (cf. Figure S6D), which was assigned to the direct H-atom abstraction from the α-carbon atom by HO^●^ radicals (see path 3 in Scheme 3). This value is reasonable considering in this case a rather low C^α^-H bond energy [27]. A quite surprising result is the short lifetime of **αC^●^** radicals which decays via a mono-exponential process with the rate constant *k* = 1.4 × 10^6^ s^−1^ (cf. Figure S6D). We tentatively suggest that by-products are formed by unimolecular decay due to β-fragmentation with formation of the acyl radical, CH_3_C(O)^•^ [28].

The pseudo-first order growth *k* = 3.7 × 10^5^ s^−1^ of the **SN^●^** concentration can be assigned to the overall reaction with the first step leading to S^●+^, followed by the concerted cyclization and deprotonation (see Scheme 3). The decay of the SN^●^ radicals is rather slow and occurs with the pseudo-first order rate constant *k* = 8.2 × 10^3^ s^−1^. Based on earlier results [14,15], protonation of the **SN^●^** radicals provides the most facile mechanistic reaction pathway of their decay. On the basis of the extracted concentration profiles of the **SN^●^** radicals in cyclic L-Met-L-Met at various pHs, it was possible to evaluate the rate constant of SN^●^ radicals with protons to be 2.1 × 10^9^ M^−1^s^−1^ [15]. At pH 7, these reactions occur with the pseudo-first order rate constant 2.1 × 10^2^ s^−1^, which are much lower than the pseudo-first order rate constant measured for the decay of the **SN^●^** radicals formed from compound **1** (vide supra). Therefore, the most probable pathway responsible for the decay of the **SN^●^** involves *N*-protonation (e.g., by water molecules), followed by deprotonation at the αC-carbon leading to **αC^●^** radicals (cf. Scheme 3). These reactions provide an irreversible entry to these C-centered radicals and were previously suggested for linear peptides containing Met residue [14]. Since **αC^●^** radicals are formed in a slow process, this fact, combined with their very short lifetime, rationalizes their absence in the resolved absorption spectra recorded at longer times.

### 2.2. γ-Radiolysis and Product Analysis

In addition to the reactive species e^−^_aq_, HO^•^, and H^•^, radiolysis of neutral water leads also to H^+^ (0.28) and H_2_O_2_ (0.07); in parenthesis the *G* in µmol J^−1^ [7]. In N_2_O-saturated solution, the *G*(HO^•^) = 0.56 µmol J^−1^, therefore HO^•^ and H^•^ account for 90% and 10%, respectively, of the reactive species (cf. Reactions (1) and (2)).

**Figure 3:**
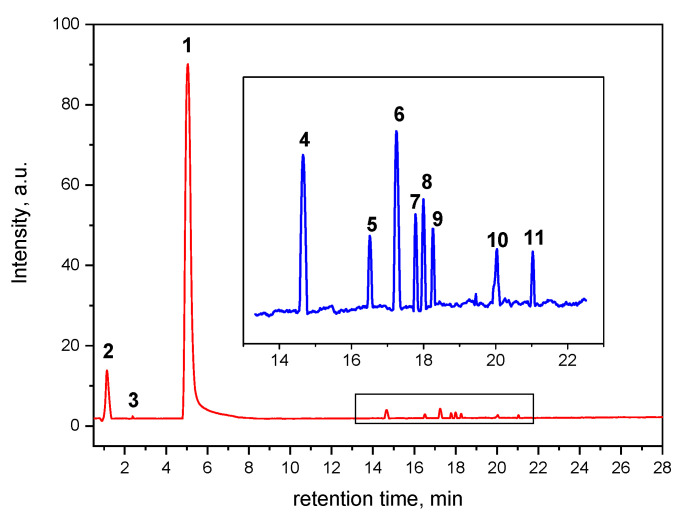
HPLC run of γ-irradiation of N_2_O-saturated aqueous solutions of 1.0 mM *N*-acetyl methionine methyl ester (**1**) at natural pH at a dose of 800 Gy (dose rate of 46.7 Gy min^−1^). The consumption of **1** led to the formation of 10 products. Inset: expansion of the chromatogram between 14 and 22 min.

N_2_O-saturated solutions containing compound **1** (1.0 mM) at natural pH were irradiated for 400 and 800 Gy under stationary state conditions with a dose rate of 46.7 Gy min^−1^ followed by LC–MS and high-resolution MS/MS analysis. A representative LC-MS analysis of the 800 Gy irradiated sample is shown in Figure 3.

**Scheme 4:**
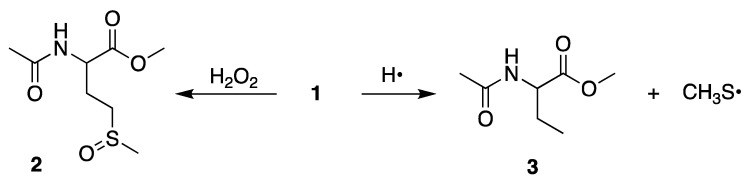
The reaction of **1** with H_2_O_2_ and H^•^ atom affords sulfoxide **2** and α-aminobutyric **3** derivatives, respectively.

Eleven compounds were detected in the chromatogram including the starting material **1**. All peaks were identified and their chemical structures assigned by examination of their high-resolution mass data and characteristic fragmentation patterns (see Figures S7 and S8). In Scheme 3 and Scheme 4 the structures of all products are reported that will be described in some detail below.

Combination of the pulse radiolysis results on reactive intermediates and the structural information obtained from the high-resolution MS/MS allows the depiction the mechanistic proposal of compound **1** transformation by γ-radiolysis (Scheme 3). It is well documented from previous studies on Met derivatives that the sulfoxide **2** is formed due to the in-situ generation of hydrogen peroxide [17,19], while the H^•^ addition to the sulfur (*k* = 1.7 × 10^9^ M^−1^s^−1^) with the formation of a sulfuranyl radical intermediate affords compound **3** and CH_3_S^•^ radical (Scheme 4) [29,30].

From the pulse radiolysis studies described above, the reaction of HO^•^ with **1** (*k* = 1.1 × 10^10^ M^−1^s^−1^) followed one main and two minor paths. In Scheme 3, the formation of adduct radical (**HOS^•^**) is the main path (path 1), while the two minor ones are: the H-atom abstraction from the CH_2_-S-CH_3_ moiety to give the intermediate **αS^•^** radicals (path 2) and the H-atom abstraction from the N-CH-CO moiety to give the intermediate **αC^•^** radical (path 3). Table 1 shows that 1.1 μs after the pulse the distribution percentages were 73.6, 1.9 and 11.35% for **HOS^•^**, **αS^•^** and **αC^•^**, but at 6 μs after the pulse the relative contribution changed to 5.3, 45.6 and 3.5%, respectively. The **HOS^•^** follows a first-order decay (*k*_d_ = 5.6 × 10^5^ s^−1^) by HO^−^ elimination to give the sulfide radical cation, which is at the crossroad of various possible reactions affording the intermediates **SS^•+^**, **αS^•^**, **SN^•^** and **αC^•^**. We recall from pulse radiolysis section that the radiation chemical yields of **HOS^•^** and the sum of radiation chemical yields of **SS^•+^**, **αS^•^** and **SN^•^** species at 3 μs and 6 μs after the pulse (Table 1) suggest that the formation of **SS^•+^**, **αS^•^** and **SN^•^** species follows the decay of **HOS^•^**, as discussed in the previous section.

We suggest that the disulfide radical cation (**SS^•+^**), which is in equilibrium with sulfide radical cation (S^•+^) and starting material, fragments and affords the observed disulfide **5** [17]. Moreover, the S^•+^ is prompt to deprotonation. Evidence from the pulse radiolysis experiments indicated that **SN^•^** radical is the progenitor of **αC^•^** radical and the proposed mechanism is suggested in Scheme 3, including the tentatively suggested unimolecular decay by β-fragmentation with formation of acyl radical [28]. We do not have confirmation of this latter pathway by the present LC-MS data.

Lastly, the **αS^•^** radicals, which can be both **αS(1)^•^** and **αS(2)^•^** are shown in Scheme 3. Table 1 reports the percentage contribution as a function of time, i.e., 1.9, 30.3 and 45.6% for 1.1, 3 and 6 μs after the pulse, which means that at longer time scale the **αS^•^** radicals are the only remaining reactive species together with CH_3_S^•^ radical derived from the H^•^ atom reactivity (cf. Scheme 4). The cross-termination of **αS(1)^•^** and **αS(2)^•^** with CH_3_S^•^ affords compounds **4** and **6**, respectively (see Scheme 3).

The data from the high-resolution MS/MS showed that compounds **7**, **8**, **9**, **10** and **11** are dimers of **αS^•^** radicals (Scheme 3). Figure S8 shows the high-resolution MS/MS spectra of the five compounds. The accurate masses of these products, *m*/*z* 409.1484, 409.1480, 409.1480, 409.1479, 409.1478, correspond to a molecular weight MH^+^ equivalent of two **αS^•^** radicals. Although the fragmentation patterns are not diagnostic, some information can be extracted. All of them show that initial fragmentations with a loss of CH_4_O, CH_4_S, C_2_H_4_O and/or C_2_H_2_O.

**Figure 4:**
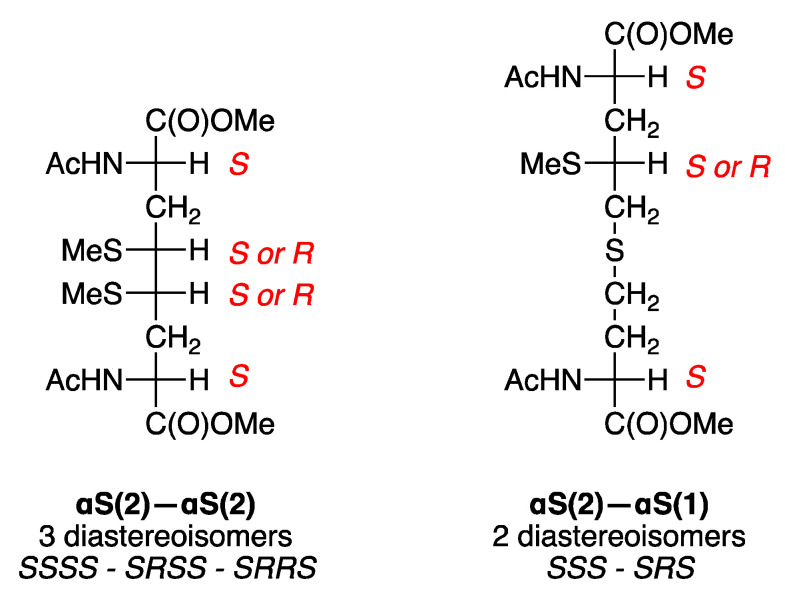
The chemical structures of **αS(2)–αS(2)** and **αS(2)–αS(1)** indicating the configuration of stereocenters.

Further structural information may be obtained from the analysis of potential diasteroisomers. What is the ratio of the two **αS^•^** radicals? It is expected **αS(2)^•^** radical to be in higher concentration than **αS(1)^•^** and in line with the higher stability of secondary vs. primary alkyl radical due to favorable deprotonation from the precursor sulfide radical cation. Assuming that the concentration of **αS(2)^•^** radical is twice that of the **αS(1)^•^** radical, it is expected to having αS(2)–αS(2) and αS(2)–αS(1) from a probability point of view of termination steps. Figure 4 shows that αS(2)–αS(2) has four stereocenters, two are from the starting material fixed at the S configuration whereas two new stereocenters generated from the self-termination can be *R* or *S*. In total four products, two of them are identical, and therefore we expect to have *SSSS*, *SRSS* and *SRRS* diastereoisomers.

Figure 4 shows that αS(2)–αS(1) has three stereocenters, two are from the starting material fixed at the *S* configuration and one stereocenter is generated from the cross-termination of the two radicals, producing the diastereoisomers *SSR* and *SRS*. In total, five diastereoisomers that we associated with the five dimeric products detected by LC-MS in γ-radiolysis of the compound **1**.

### 2.3. Photosensitized Oxidation by ^3^CB

**Figure 5:**
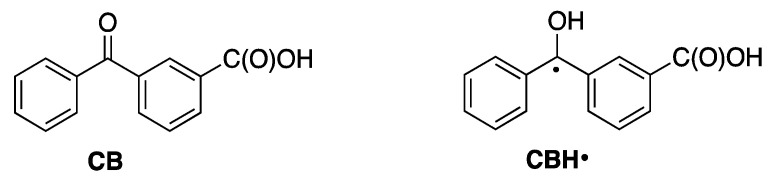
Structures of the sensitizer (3-carboxybenzophenone, CB) and its respective photo-reduction product (ketyl radical, CBH^•^).

Sensitized by triplet CB (^3^CB) oxidation of Met derivatives leads to numerous transients, well characterized in our earlier studies [23,25,31]. Laser flash photolysis (LFP) results of the ^3^CB with **1** are presented in Figure S9. The main species observed are ^3^CB and ketyl radical (**CBH^•^**), with a minor contribution of radical anion (CB^•−^) (Figure 5). As expected, the transient species derived from compound **1** could not be directly observed due to the strong absorption overlap of the transients derived from CB photochemistry. However, it is expected that the radical coupling reactions derived from the two **αS^•^** radicals are predominant.

**Figure 6:**
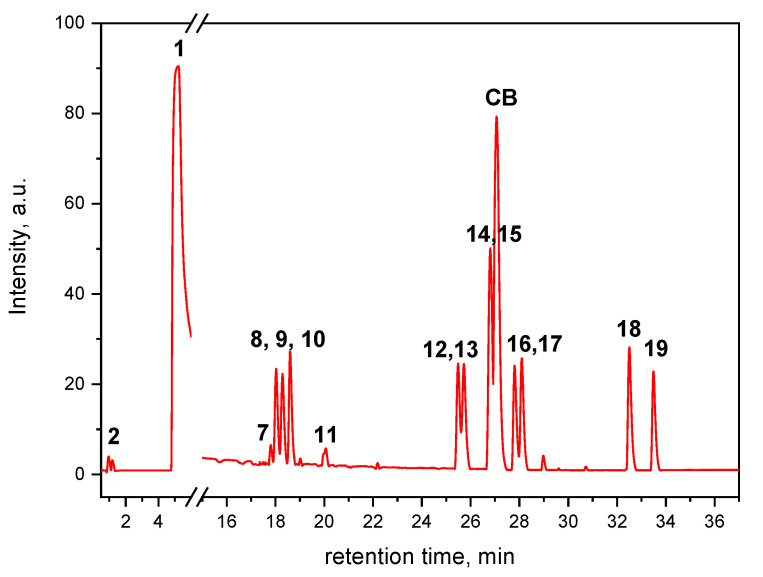
HPLC run of 355 nm irradiated solution containing **CB** (4 mM) and *N*-acetyl methionine methyl ester (**1**) (20 mM). The peaks are labelled with numbers referring to products shown in Scheme 5 and Figure 7.

**Scheme 5:**
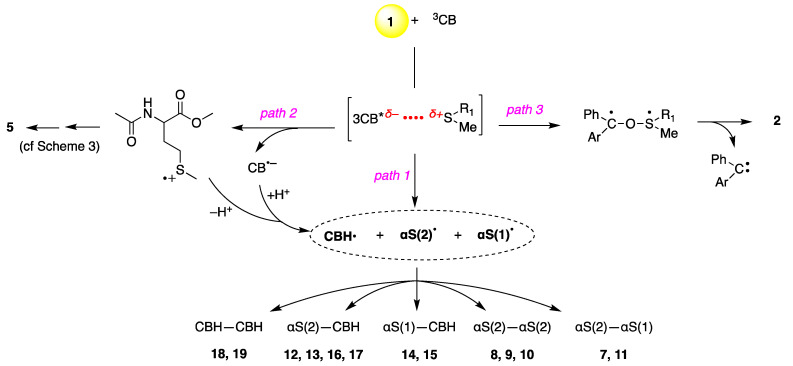
Proposed mechanism for the reaction of 3CB triplet with *N*-acetyl methionine methyl ester (**1**) at natural pH 7.

The Ar-saturated solutions containing CB (4 mM) and compound 1 (20 mM) at natural pH were irradiated for 20 min using a CW 355 nm laser (50 mW, see Experimental Section for details). A representative LC-MS analysis of the irradiated solution is shown in Figure 6. A variety of compounds were detected in the chromatogram, including the starting material **1** and **CB**. The high-resolution mass data and fragmentation patterns of all peaks were analyzed and information on their chemical structures were extracted. In Scheme 5, the structures of major products are reported that will be described in some detail in the following paragraphs.

Based on LFP results for the reactive intermediates and the structural information obtained from the high-resolution MS/MS, the mechanistic proposal of compound **1** transformation by photolysis can be depicted (Scheme 5). The formation of a complex between ^3^CB and **1** through its sulfur atom is well documented followed by the H-atom abstraction as the main pathway, yielding the two **αS(1)^•^** and **αS(2)^•^** radicals (path 1) [25,31,32]. Evidence that a small portion of the complex undergoes one electron transfer, with the formation of sulfide radical cation (S^●+^), was obtained (path 2). In analogy with the above-described mechanism in the radiolysis section, we detected traces of compound **5** suggesting a small contribution to the formation of **αS(1)^•^** and **αS(2)^•^** radicals. Moreover, we detected traces of sulfoxide **2**. We speculate that the oxidation of sulfur is due the formation of a biradical and its further fragmentation to the corresponding sulfoxide (path 3). The fate of **αS^•^** radicals will depend on the relative concentration of **αS(1)^•^** and **αS(2)^•^** and the presence of a **CBH^•^** radical.

**Figure 7:**
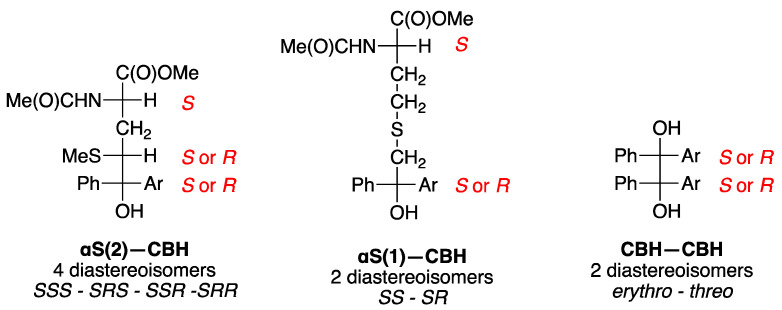
The chemical structures of αS(2)–CBH, αS(1)–CBH and CBH–CBH) indicating the configuration of stereocenters.

The HPLC run in Figure 6 shows compounds in three retention time intervals:

(i) In the interval of 17.5–19.5 min there are three major peaks that correspond to compounds **8**, **9** and **10**, and 2 minor peaks that correspond to compounds **7** and **11**, which all are the dimers of **αS^•^** radicals observed in γ-radiolysis experiments (cf. Scheme 3). It is worth underlining that the accurate masses of these products and the fragmentation patterns are identical in all sets of experiments (Figure S10). We assigned the structures **8**, **9** and **10** to the 3 diastereoisomers of the αS(2)–αS(2) dimers and **7** and **11** (minor peaks) to the 2 diastereoisomers of αS(2)–αS(1) reported in the previous radiolysis section (see Scheme 5).
(ii) In the interval of 32–34 min there are two peaks that are individuated as compounds **18** and **19** (Figure 6). Their accurate masses (*m*/*z* 455.1527, and 455.1522) correspond to the MH^+^ of the dimer CBH–CBH (Figure S11). Figure 7 shows that CBH–CBH has two stereocenters and a plane of symmetry that correspond to *erythro* and *threo* diastereoisomers.
(iii) In the interval of 25–29 min there is the major peak that corresponds to **CB**, with two doublets on the right and left sides, respectively, and one singlet in the shoulder of **CB** (Figure 6). In this area of HPLC run there are the cross-coupling products of **αS^•^** and **CBH^•^** radicals. The accurate masses of two couples of compounds named **12**, **13** (*m*/*z* 414.1391, 414.1393) and **16**, **17** (*m*/*z* 414.1390, 414.1393), as well as their fragmentation patterns, are identical and assigned to αS(2)–CBH (Scheme 5 and Figure S12). Figure 7 shows that αS(2)–CBH has three stereocenters, one is from the starting material fixed at the *S* configuration and two stereocenters are generated from the cross-termination of the two radicals, producing the diastereoisomers *SSS*, *SRS*, *SSR* and *SRR*. Regarding the singlet in the shoulder of **CB**, having also *m*/*z* 414.1392, but different fragmentation patterns, it is assigned to αS(1)–CBH (Figure S13). As shown in Figure 7, this compound has two stereocenters, the usual *S* configuration from the starting material and a new one generated from the cross-termination of the two radicals, producing the diastereoisomers *SS* and *SR.* It is likely that, under our HPLC conditions, the two diastereoisomers be under the same peak, or one of them overlap with **CB**.

The above analysis suggests that the concentration of **αS(2)^•^** radical is much higher than **αS(1)**^•^, as expected from the competition of the two H-atom abstraction steps with formation of secondary vs. primary alkyl radical, being the difference in BDE energy 2–3 kcal/mol. The LC run (Figure 6) together with the proposed mechanism (Scheme 5) suggests a ratio of 5–5.5 between the two **αS^•^** radicals. In this situation, the intermediates **CBH^•^** and **αS(2)^•^** will be the main players in the self-termination steps with formation of **18**, **19** and **8**, **9**, **10**, respectively, as well as in the cross-termination reaction with formation of **12**, **13**, **16** and **17** diastereoisomers.

## 3. Materials and Methods

### 3.1. Pulse Radiolysis

The pulse radiolysis experiments were performed with the LAE-10 linear accelerator at the Institute of Nuclear Chemistry and Technology in Warsaw, Poland with a typical electron pulse length of 10 ns and 10 MeV of energy. A detailed description of the experimental setup has been given elsewhere along with basic details of the equipment and its data collection system [33,34]. The 1 kW UV-enhanced xenon arc lamp (Oriel Instruments, Stratford, CT, USA) was applied as a monitoring light source. The respective wavelengths were selected by MSH 301 monochromator (Lot Oriel Gruppe, Darmstadt, Germany) with a resolution of 2.4 nm. The intensity of analysing light was measured by means of PMT R955 (Hamamatsu, Hamamatsu City, Shizuoka, Japan). A signal from detector was digitised using a Le Croy WaveSurfer 104MXs-B (1 GHz, 10 GS/s) oscilloscope and then send to PC for further processing. A water filter was used to eliminate near IR wavelengths.

Absorbed doses per pulse were on the order of 11 Gy (1 Gy = 1 J kg^−1^). Experiments were performed with a continuous flow of sample solutions using a standard quartz cell with optical length 1 cm at room temperature (~22 °C). Solutions were purged for at least 20 min per 250 mL sample with N_2_O before pulse irradiation. The *G*-values were calculated from the Schuler formula (Equation (3)) [35]

(3) $$G\left(S^{●}\right)=0.539+0.307\frac{\sqrt{19.6\left[S\right]}}{1+\sqrt{19.6\left[S\right]}}$$

where [S] is the HO^●^-scavenger concentration and with respect to the current work, concentration of Ac-Met-OMe. This form of the Schuler formula gives *G*(S^●^) in units of μmol J^−1^, and with respect to the current work, [S] = 0.2 mM gives *G*(S^●^) = 0.557 μmol J^−1^ where (S^●^) corresponds to all radicals formed in the system. The dosimetry was based on N_2_O-saturated solutions of 10^−2^ M KSCN which following radiolysis, produces (SCN)_2_^●−^ radicals that have a molar absorption coefficient of 7580 M^−1^cm^−1^ at *λ* = 472 nm and are produced with a yield of *G* = 0.635 µmol J^−1^ from Equation (3) [36].

### 3.2. Spectral Resolutions of Transient Absorption Spectra

The observed absorption spectra monitored at various time delays following the electron pulse, were transformed from A(*λj*) to *Gε*(*λj*) by multiplying A(*λj*) by the factor (F) from the dosimetry described in Section 3.1. A(*λj*) represents the absorbance change of the composite spectrum and F = *ε*_472_ × *G*(SCN)_2_^●−^/A_472_ where *ε*_472_ is the molar absorption coefficient of (SCN)_2_^●−^ at 472 nm and *G*(SCN)_2_^●−^ is the radiation chemical yield of the SCN)_2_^●−^ (see Section 3.1) and A_472_ represents the observed absorbance change in the thiocyanate dosimeter. The optical spectra thus converted were resolved into specific components (representing individual transients) by linear regression according to the following Equation (4)

(4) $$G\varepsilon \left(\lambda_{i}\right)=\underset{j}{\sum }\varepsilon_{j}\left(\lambda_{i}\right)G_{j}$$

where *ε_j_* is the molar absorption coefficient of the *j*th species and the regression parameters, *G_j_*, are equal to the radiation-chemical yield of the *j*th species. The sum in Equation (4) is over all radical species present. For any particular time delay of an experiment, the regression analysis included equations such as Equation (4) for each *λ_i_* under consideration. Further details of this method were described elsewhere [15].

The reference spectra of these transients were previously collected and applied in the spectral resolutions (cf. Figure S2 in Supplementary Materials) [15,17]. The molar absorption coefficients of the relevant transients, which will be further identified below, are provided in the following: **HOS^●^**, *λ*_max_ = 340 nm and *ε*_340_ = 3400 M^−1^ cm^−1^; **αC^●^**, *λ*_max_ = 270 nm and *ε*_270_ = 6200 M^−1^ cm^−1^ and *λ*_max_= 370 nm and *ε*_370_ = 1800 M^−1^ cm^−1^; **αS^●^**, *λ*_max_ = 290 nm and *ε*_290_ = 3000 M^−1^ cm^−1^; **SS^●+^**, *λ*_max_ = 480 nm and *ε*_480_ = 6880 M^−1^ cm^−1^; **SN^●^**, *λ*_max_ = 390 nm and *ε*_390_ 4500 M^−1^ cm^−1^.

For the compound (**1**) studied in this work, it was not possible to generate these radicals selectively since they undergo fast mutual transformation. Based on our earlier experience, however, we can say with certainty that the changes in molar absorption coefficients within the same type of radicals are not significant and are in the limit of 15% error (5% variation in the experimental data and 10% combined error in the reported molar absorption coefficients for the UV-vis spectra of the intermediates under consideration). The fact that the combined yields of the transient species derived from their respective molar absorption coefficients are at the short time delays equal to the expected initial yield of the scavenged ^●^OH radicals by **1**, i.e., 0.56 mol J^−1^ (based on Schuler’s formula (Equation (3) in Section 3.1)), and they also never exceed this value, supports additional validation of the spectral resolutions and eliminates unreasonable fits.

Involvement of the H^●^ atom reaction is not reflected in the resolved transient spectra since the forming CH_3_S^●^ (see Scheme 4) is formed with a very low radiation chemical yield (<0.06 mol J^−1^) and is characterised by the very low molar absorption coefficient (<500 M^−1^cm^−1^). However, the involvement of H^●^ atoms is reflected in the formation of final products **4** and **6** (see Scheme 3).

### 3.3. Steady-State γ-Radiolysis

Irradiations were performed at room temperature using a ^60^Co-Gammacell at different dose rates. The exact absorbed radiation dose was determined with the Fricke chemical dosimeter, by taking *G*(Fe^3+^) = 1.61 μmol J^−1^ [37].

### 3.4. Laser Flash Photolysis

The laser flash photolysis (LFP) setup used in this work has been described in detail elsewhere [25,31]. Briefly, this setup employs Nd:YAG laser (Spectra Physics, Mountain View, CA, USA, model INDI 40-10) with 355 nm excitation wavelength as light pump and 150 W pulsed xenon (Applied Photophysics, Surrey, UK) to probe the excited sample. A flash photolysis experiment was performed in oxygen-free environment in 1 × 1 cm rectangular quartz fluorescence cells. Kinetic traces were recorded between 370 and 750 nm at 10 nm intervals. The sample contained the quencher-compound **1** (20 mM) and the sensitizer CB (4 mM) at pH = 7.

### 3.5. Steady-State Photolysis

Steady-state photochemical irradiation experiments were performed in a 1 × 1 cm rectangular cell on an optical bench irradiation system using a Genesis CX355STM OPSL laser from Coherent (Santa Clara, CA, USA) with 355 nm emission wavelength (the output power used was set at 50 mW).

### 3.6. LC-MS/MS Measurements

The LC-MS measurements were carried out using a liquid chromatography Thermo Scientific/ Dionex Ultimate 3000 system equipped with C18 reversed-phase analytical column (2.6 μm, 2.1 mm × 100 mm, Thermo-Scientific, Sunnyvale, CA, USA). The LC method employed a binary gradient of acetonitrile and water with 0.1% (*v*/*v*) formic acid. Separation was achieved with a gradient of 7–60% of acetonitrile at a flow rate of 0.3 mL/min for 42 min. This UHPLC system was coupled to a hybrid QTOF mass spectrometer (Impact HD, Bruker Daltonik, Bremen, Germany). The ions were generated by electrospray ionization (ESI) in positive mode. MS/MS fragmentation mass spectra were produced by collisions (CID, collision-induced dissociation) with nitrogen gas in the Q2 section of the spectrometer.

## 4. Conclusions

Summarizing the role of various transient species obtained by the two different time-resolved techniques and their connection with the end-product formation (Scheme 3 and Scheme 5), the **αS(2)^•^** and **αS(1)^•^** radicals play an important role in both oxidation processes, although their mode of formation and relative concentration are quite different: (a) in radiolysis, the one-electron oxidation of sulfide (generated by HO^•^ addition followed by HO^−^ elimination) is followed by α-deprotonation with formation of **αS(2)^•^** and **αS(1)^•^** radicals, in an approximately 2:1 ratio; (b) in photolysis, the formation of a complex between ^3^CB and the sulfur moiety is followed by H-atom abstraction, yielding **αS(2)^•^** and **αS(1)^•^** radicals in an approximately 5:1 ratio. The final products are formed by radical-radical combination.

The herein described complete and detailed study of the oxidation mechanisms of Met residue, simulating its position in the interior of long oligopeptides and proteins, represents a significant and original contribution to the understanding of oxidation reactions in real biological systems, i.e., proteins, applicable to research in protein therapeutics. This work offers a benchmark for the identification, quantification and mechanistic determination of products derived from oxidation of methionine derivatives.

## Figures and Tables

**Table 1:** The radiation chemical yields (*G*, μmol J^−1^) of radicals and their percentage contribution (in parenthesis) to the total yield of radicals present in the reaction of HO^●^ with compound **1** at different times after electron pulse at pH 7.0 ^a^.

Time (µs)	HOS^●^	αC^●^	αS(1)^●^ + αS(2)^●^	SS^●+^	SN^●^	Total R^●^
1.1	0.39 (73.6%)	0.06 (11.3%)	0.01 (1.9%)	0.04 (7.5%)	0.03 (5.7%)	0.53
3	0.16 (28.6%)	0.02 (3.6%)	0.17 (30.3%)	0.11 (19.6%)	0.10 (17.9%)	0.56
6	0.03 (5.3%)	0.02 (3.5%)	0.26 (45.6%)	0.13 (22.8%)	0.13 (22.8%)	0.57

^a^ For a procedure of *G* determination see Section 3 and [15].

**Table 2:** Kinetic data for growth and decay of radicals present after the reaction of HO^●^ with compound **1** at pH 7.0 from the pulse radiolysis studies.

*k*, s^−1^	HOS^●^	αC^●^	αS^●^	SS^●+^	SN^●^
*k*_growth_	2.1 × 10^6^ 1.1 × 10^10 a^	1.5 × 10^6^ 7.5 × 10^9 a^	4.1 × 10^5 b^ 3.6 × 10^4 c^	4.7 × 10^5^ 2.2 × 10^9 d^	3.7 × 10^5^
*k*_decay_	5.6 × 10^5^	1.4 × 10^6^	4.0 × 10^3^	3.6 × 10^4^	8.2 × 10^3^

^a^ Τhe second-order rate constants (M^−1^s^−1^) measured based on 0.2 mM concentration of **1**; ^b^ the first-order rate constant of the fast growth; ^c^ the first-order rate constant of the slow growth; ^d^ the second-order rate constant (M^−1^s^−1^) measured based on 0.2 mM concentration of **1**, and corrected for the backward reaction of the equilibrium, cf. Scheme 3.

## Data Availability

All data are displayed in the manuscript.

## Supplementary Materials

The following are available online at https://www.mdpi.com/article/10.3390/ijms22094773/s1, Figure S1: The structures of six reactive intermediates identified in the pulse radiolysis experiments. Figure S2: Reference spectra used in the resolutions of the transient absorption spectra following ^●^OH-induced oxidation of CH_3_C(O)N-Met-OCH_3_. Figure S3: Resolution of the spectral components in the transient absorption spectrum recorded 1.1 µs after the electron pulse in N_2_O-saturated aqueous solution containing 0.2 mM AcN-Met-OMe at pH 7.0. Figure S4: Resolution of the spectral components in the transient absorption spectrum recorded 3 µs after the electron pulse in N_2_O-saturated aqueous solution containing 0.2 mM AcN-Met-OMe at pH 7.0. Figure S5: The sum of all radicals (HOS^●^, αC^●^, SS^●+^, SN^●^, αS^●^) taken in spectral resolutions as a function of time. Figure S6: First-order kinetic fits of the growth and decay of radicals HOS^●^ (panel A), SS^●+^ (panel B), αS^●^ (panel C), αC^●^ (panel D), and SN^●^ (panel E). Figure S7: High-resolution MS/MS spectra of the products **4** (*m*/*z* 252.0731) and **6** (*m*/*z* 252.0732) derived from the cross-termination of **αS(1)^•^** and **αS(2)^•^** with CH_3_S^•^ and product **5** (*m*/*z* 238.0578)–a disulfide. Figure S8: High-resolution MS/MS spectra of the five dimeric products **7** (*m*/*z* 409.1484), **8** (*m*/*z* 409.1480), **9** (*m*/*z* 409.1480), **10** (*m*/*z* 409.1479) and **11** (*m*/*z* 409.1478) derived from the combination of two **αS^•^** radicals. Figure S9: Transient absorption spectra following LFP of CB (4 mM) and *N*-AcMetOCH_3_ (20 mM) for different time delays at pH 7. Figure S10: High-resolution MS/MS spectra of the dimeric products **8** (*m*/*z* 409.1481), **9** (*m*/*z* 409.1484) and **10** (*m*/*z* 409.1480) derived from the combination of two **αS(2)^•^** radicals. Figure S11: High-resolution MS/MS spectra of the two dimeric products **18** (*m*/*z* 455.1527) and **19** (*m*/*z* 455.1522) derived from of the combination of two **CBH^•^** radicals. Figure S12: High-resolution MS/MS spectra of the products **12** (*m*/*z* 414.1391, **13** (*m*/*z* 414.1393), **16** (*m*/*z* 414.1390) and **17** (*m*/*z* 414.1393) derived from the cross-termination of **αS^•^** and **CBH^•^** radicals. Figure S13: High-resolution MS/MS spectra of the product **14** (*m*/*z* 414.1392) derived from the cross-termination of **αS^•^** and **CBH^•^** radicals.
