# Supplementary material for: Radiation- and Photo-Induced Oxidation Pathways of Methionine in Model Peptide Backbone under Anoxic Conditions
Source: Int J Mol Sci. 2021 Apr 30;22(9):4773. doi: 10.3390/ijms22094773 (PMC8125225; doi:10.3390/ijms22094773)
Supplement: Supplementary file 1 [file ijms-22-04773-s001.zip › ijms-1173808-supplementary.pdf]

Supporting Information

Radiation- and Photo-induced Oxidation Pathways of Methionine in Model Peptide Backbone Under Anoxic Conditions

Tomasz Pedzinski, Katarzyna Grzyb, Konrad Skotnicki, Piotr Filipiak, Krzysztof Bobrowski*, Chryssostomos Chatgilialoglu,* Bronislaw Marciniak,*

*Corresponding Authors: chrys@isof.cnr.it (C.C.), marcinia@amu.edu.pl (B.M.), kris@ichtj.pl (K.B.)

Contents:

Figures S1, S2 and S3	Page S2
Figures S4 and S5	S3
Figure S6	S4
Figure S7	S5
Figure S8	S6
Figure S9	S7
Figure S10	S8
Figure S11	S9
Figure S12	S10
Figure S13	S11

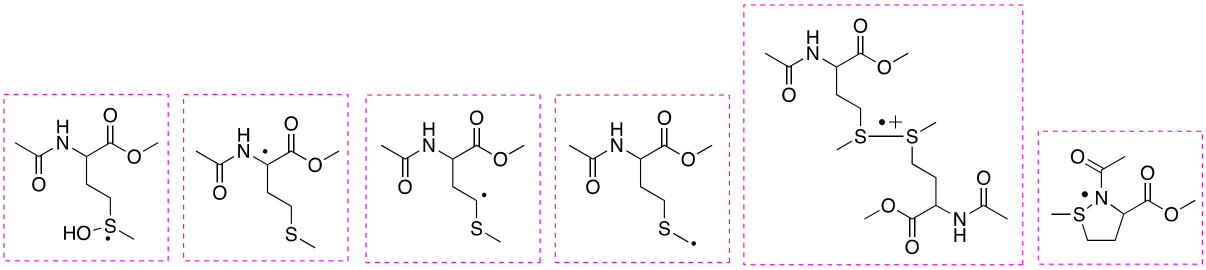

HOS• αC• αS(2)• αS(1)• SS•+ SN•

Figure S1. The structures of six reactive intermediates identified in the pulse radiolysis experiments.

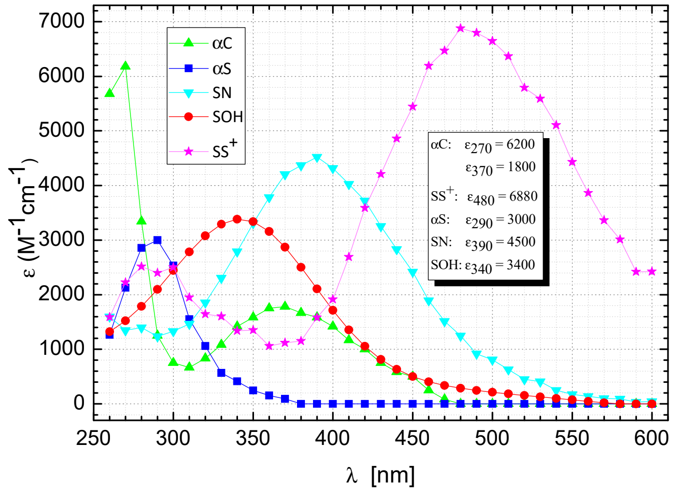

Figure S2. Reference spectra used in the resolutions of the transient absorption spectra following •OH-induced oxidation of $CH_3C(O)N$-Met-OCH_3.

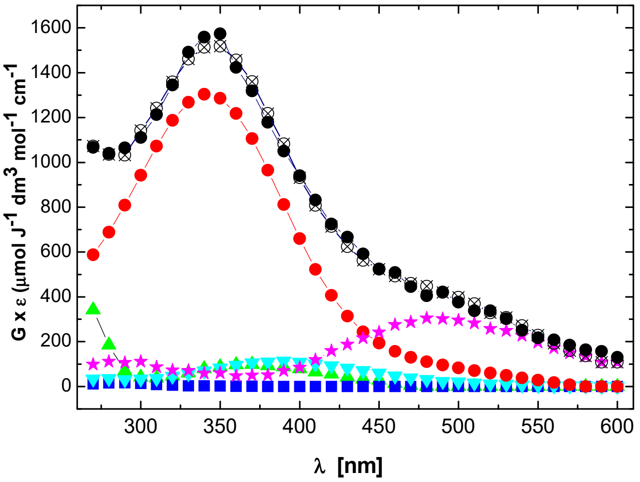

Figure S3. Resolution of the spectral components: HOS• (●), αC• (▲), αS(1)• and αS(2)• (■), SS•+ (★), SN• (▼) in the transient absorption spectrum recorded 1.1 µs (●– experimental; ⊗– fit) after the electron pulse in N_2O-saturated aqueous solution containing 0.2 mM AcN-Met-OMe at pH 7.0.

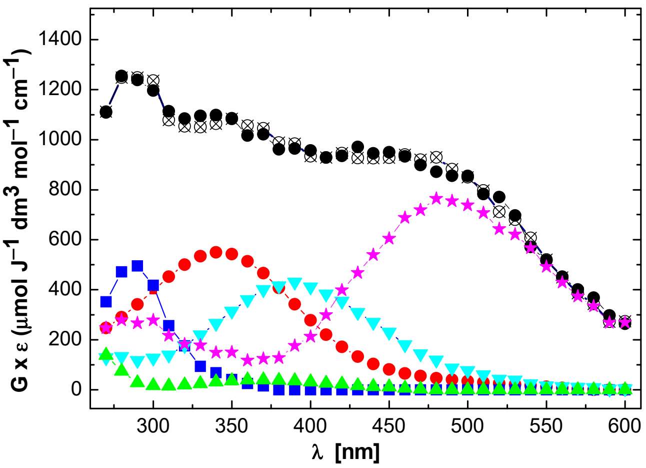

Figure S4. Resolution of the spectral components: HOS$^{\bullet}$ (●), αC$^{\bullet}$ (▲), αS(1)$^{\bullet}$ and αS(2)$^{\bullet}$ (■), SS$^{\bullet+}$ (★), SN$^{\bullet}$ (▼) in the transient absorption spectrum recorded 3 µs (●– experimental; ⊗– fit) after the electron pulse in N_2O-saturated aqueous solution containing 0.2 mM AcN-Met-OMe at pH 7.0

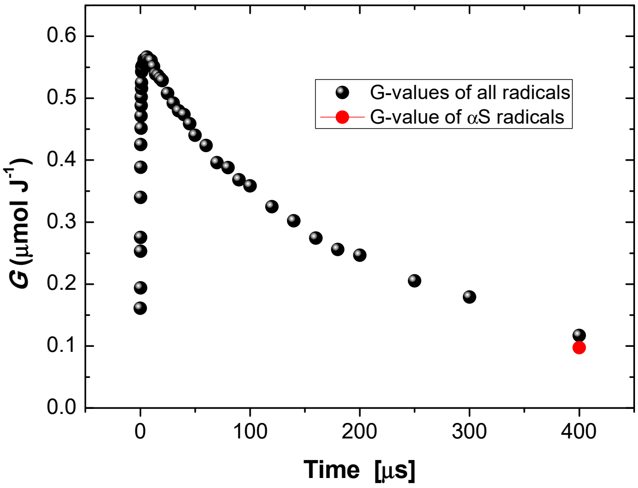

Figure S5. The sum of all radicals (HOS$^{\bullet}$, αC$^{\bullet}$, SS$^{\bullet+}$, SN$^{\bullet}$, αS$^{\bullet}$) taken in spectral resolution as a function of time.

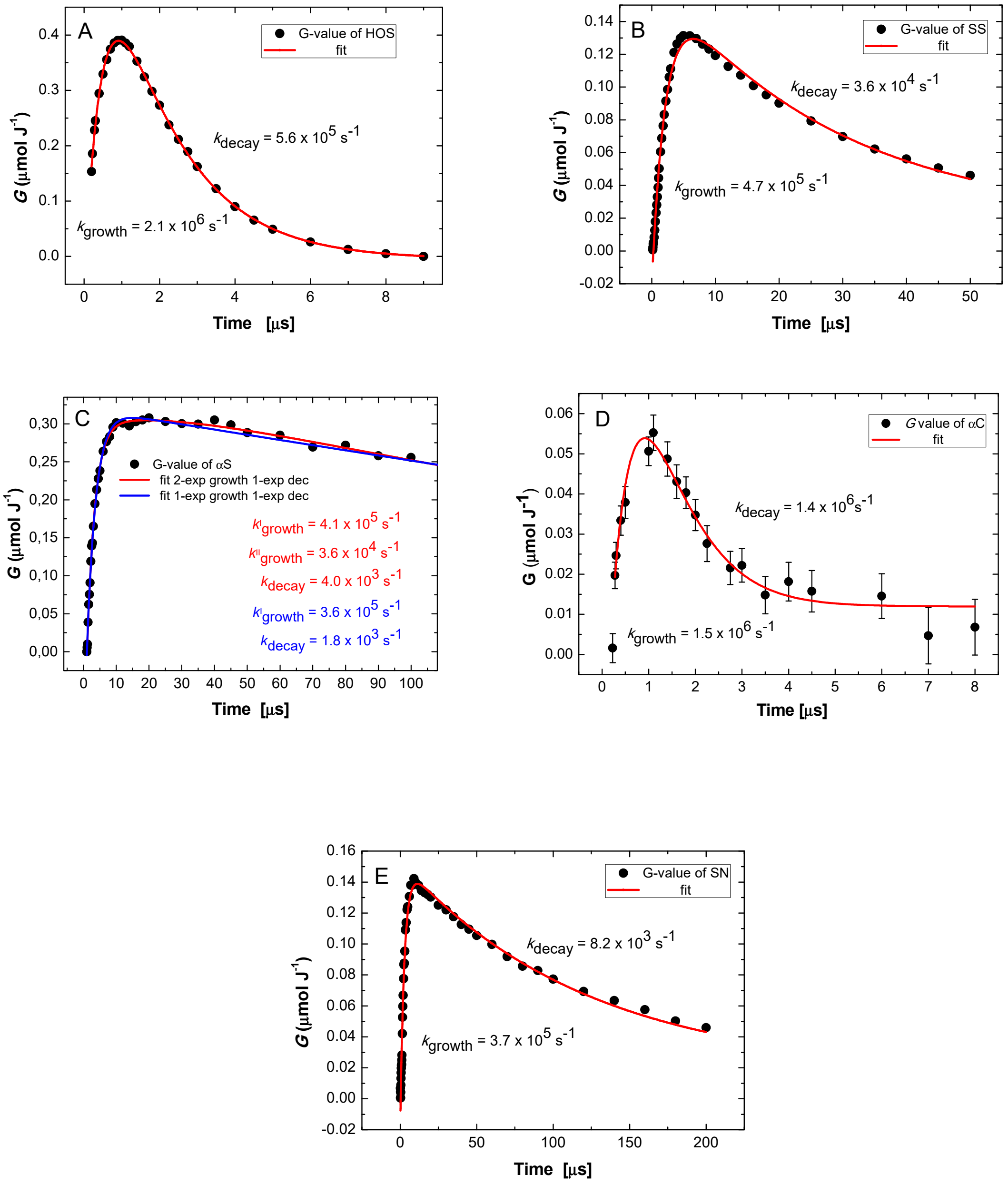

Figure S6. First-order kinetic fits of the growth and decay of radicals $HOS^{\bullet}$ (panel A), $SS^{\bullet +}$ (panel B), $\alpha S^{\bullet}$ (panel C), $\alpha C^{\bullet}$ (panel D), and $SN^{\bullet}$ (panel E).

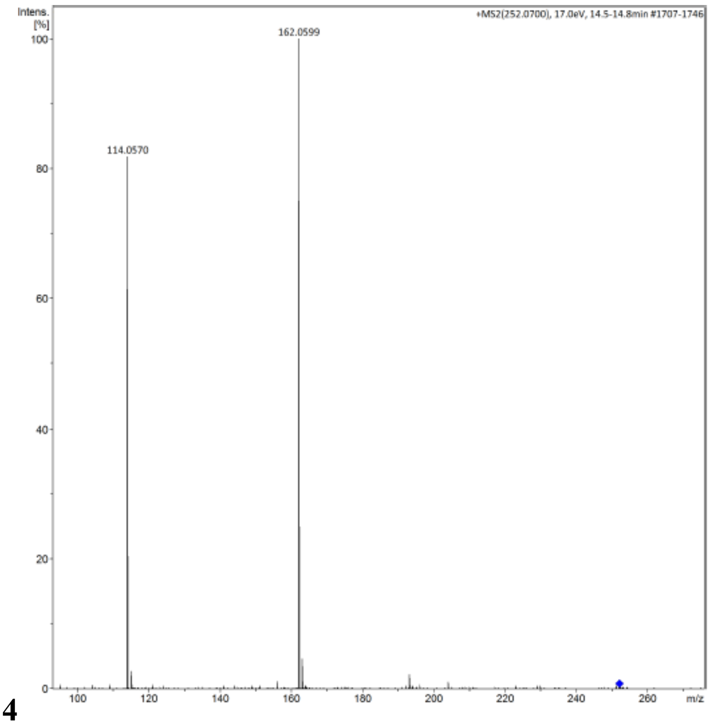

Compound **4**

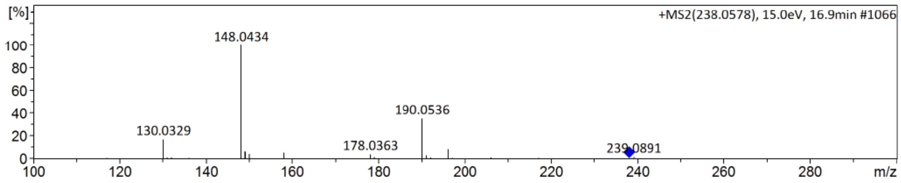

Compound **5**

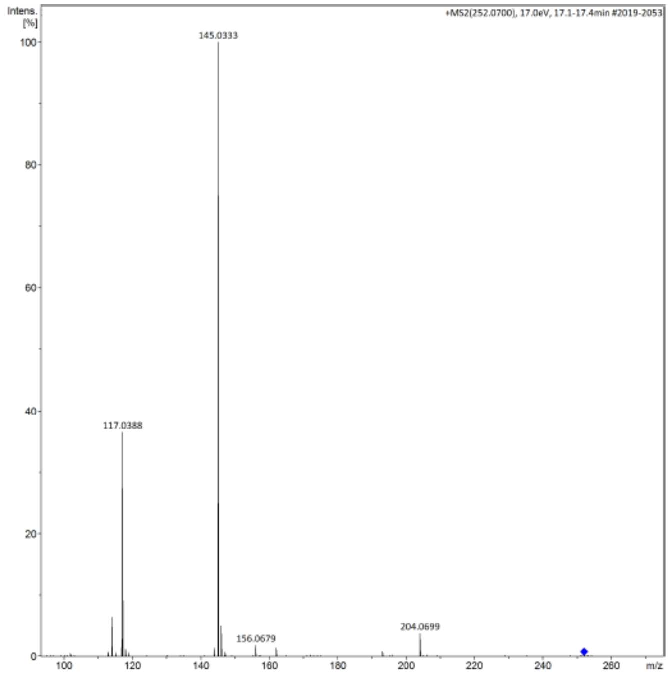

Compound **6**

Figure S7. High-resolution MS/MS spectra of the products **4** (*m/z* 252.0731) and **6** (*m/z 252.0732*) derived from the cross-termination of **αS (1)˙** and α**S(2)˙** with $CH_3S^{\cdot}$ and product **5** (m/z 238.0578) – a disulfide.

Compound **7**

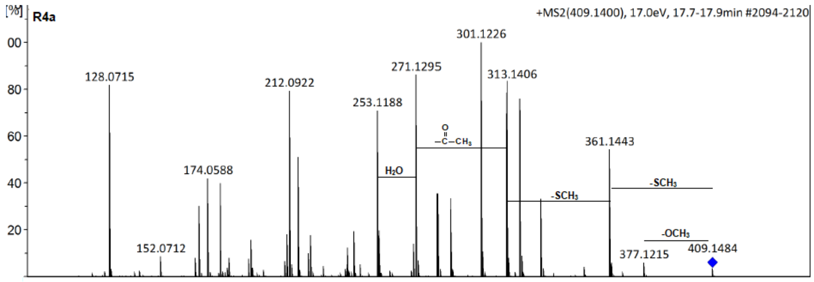

Compound **8**

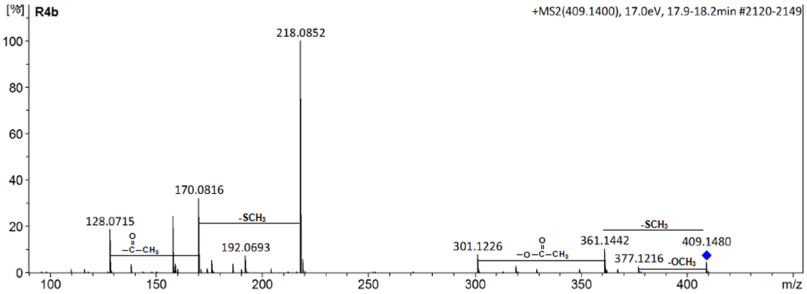

Compound **9**

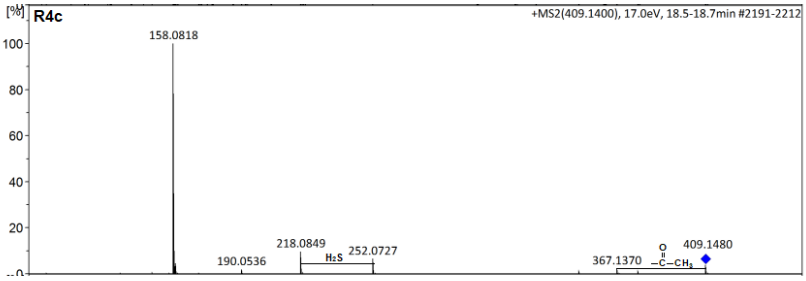

Compound **10**

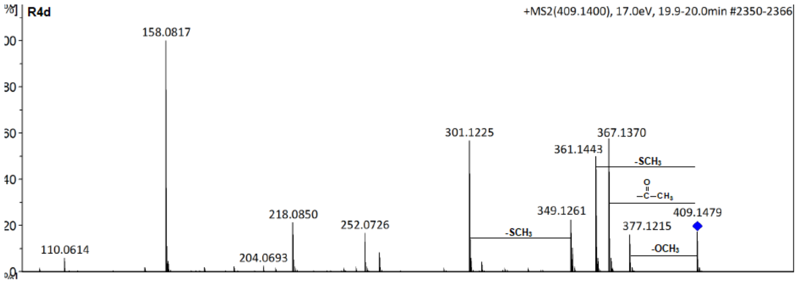

Compound **11**

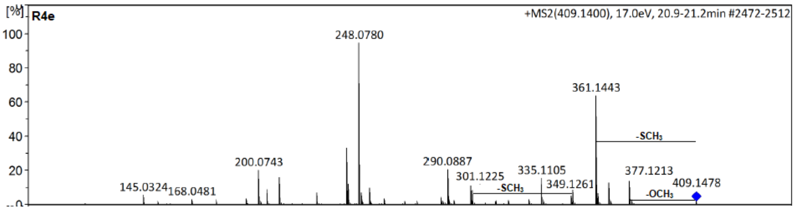

Figure S8. High-resolution MS/MS spectra of the five dimeric products **7** (*m/z* 409.1484), **8** (*m/z* 409.1480), **9** (*m/z* 409.1480), **10** (*m/z* 409.1479) and **11** (*m/z* 409.1478) derived from the combination of two $\alpha S^{\bullet}$ radicals.

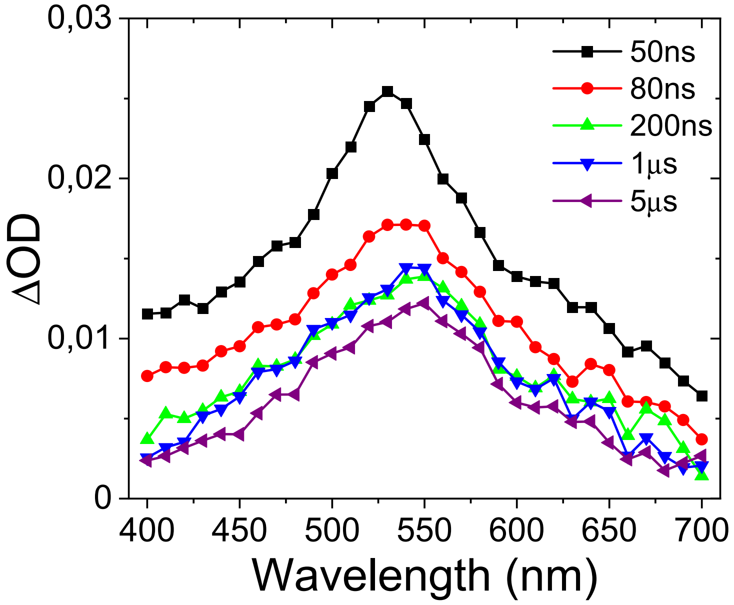

Figure S9. Transient absorption spectra following LFP of CB (4mM) and *N*-AcMetOCH$_3$ (20mM) for different delay times at pH 7. The initially formed CB excited triplet with a transient absorption maximum at 520 nm (50 ns delay time) yields ketyl radical CBH$^{\bullet}$ with a maximum at 550 nm (observed at 1-5 μs timescale).

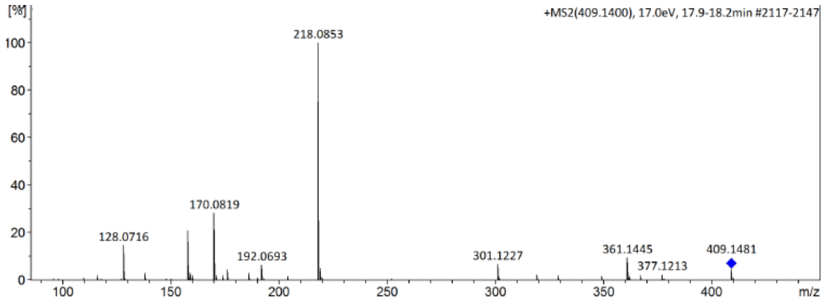

Photolysis 8

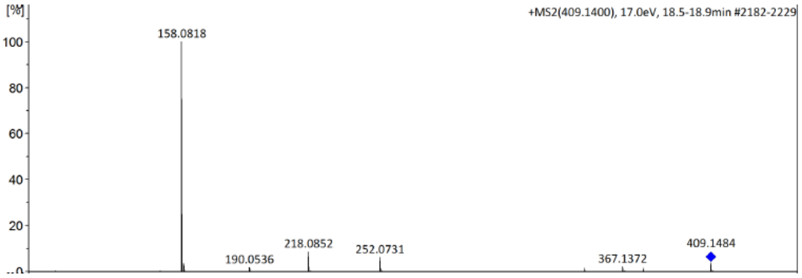

Photolysis 9

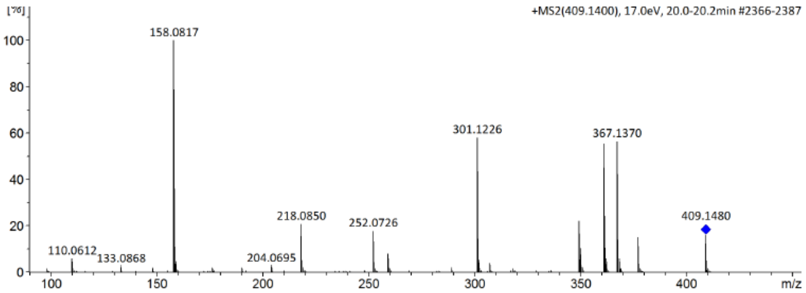

Photolysis 10

Figure S10. High-resolution MS/MS spectra of the dimeric products **8** (*m/z* 409.1481), **9** (*m/z* 409.1484) and **10** (*m/z* 409.1480) derived from the combination of two **αS(2)$^{\bullet}$** radicals.

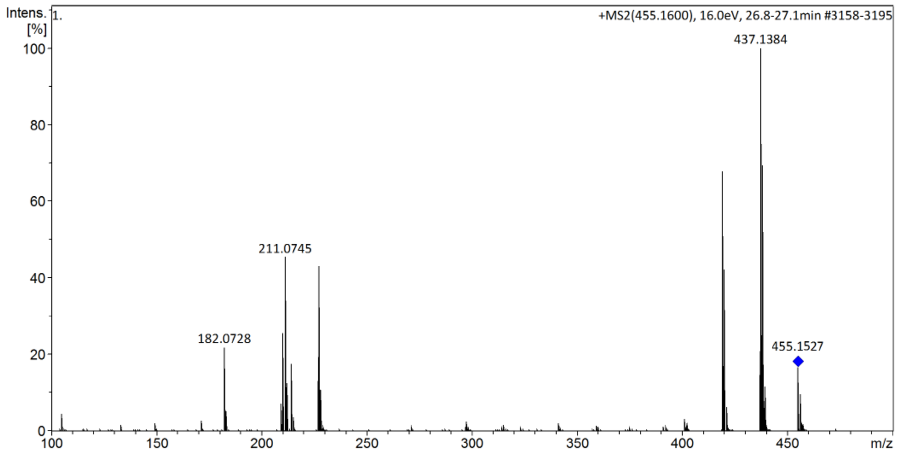

Photolysis 18

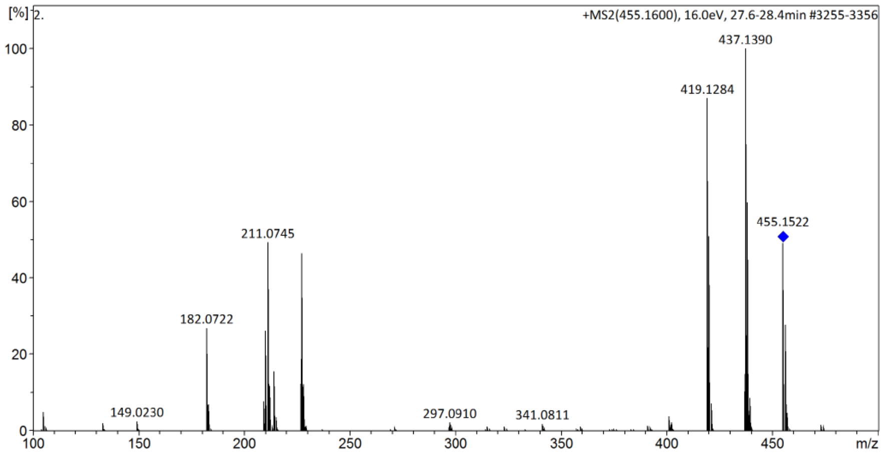

Photolysis 19

Figure S11. High-resolution MS/MS spectra of the two dimeric products **18** (*m/z* 455.1527) and **19** (*m/z* 455.1522) derived from of the combination of two **CBH**$^{\bullet}$ radicals.

Photolysis 12

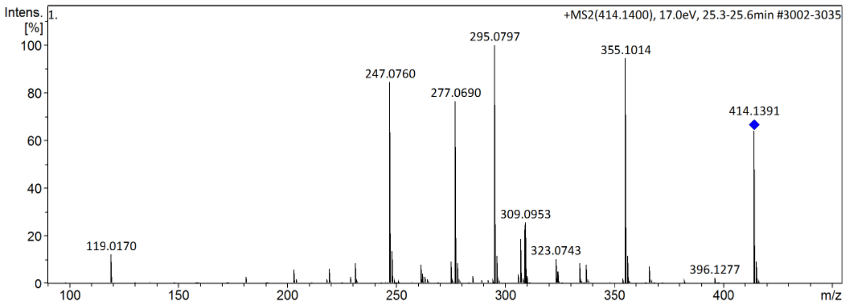

Photolysis 13

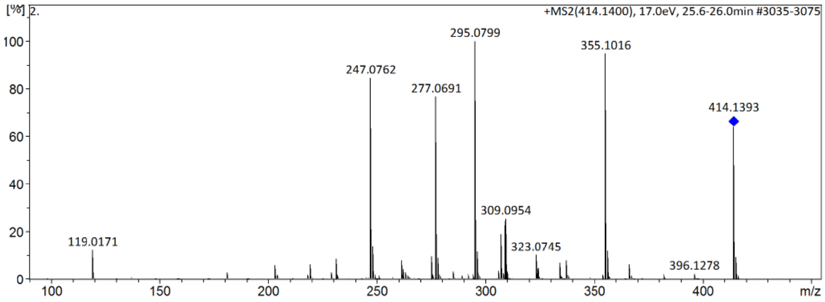

Photolysis 16

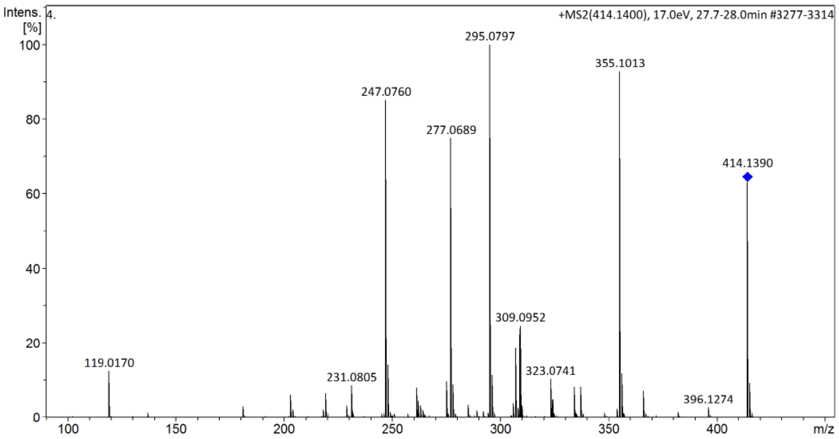

Photolysis 17

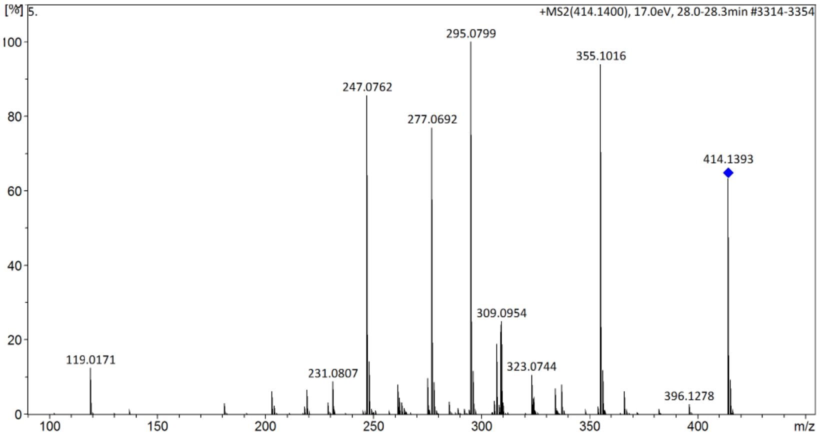

Figure S12. High-resolution MS/MS spectra of the products **12** (*m/z* 414.1391, **13** (*m/z* 414.1393), **16** (*m/z* 414.1390) and **17** (m/z 414.1393) derived from the cross-termination of α**S**• and **CBH**• radicals.

Photolysis 14

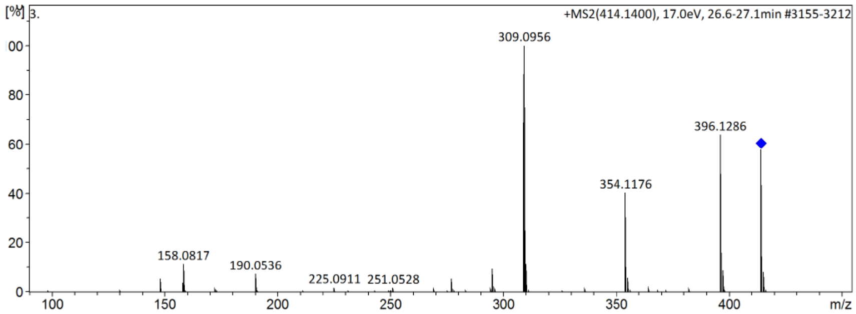

Figure S13. High-resolution MS/MS spectra of the product **14** (m/z 414.1392) derived from the cross-termination of α**S**$^•$ and **CBH**$^•$ radicals.
